# *N*-(1-Acetyl-*r*-7,*c*-9-diphenyl-4,8-dithia-1,2-diaza­spiro­[5.4]dec-2-en-3-yl)acet­amide

**DOI:** 10.1107/S1600536808001025

**Published:** 2008-01-23

**Authors:** D. Gayathri, D. Velmurugan, S. Umamatheswari, S. Kabilan, K. Ravikumar

**Affiliations:** a Centre of Advanced Study in Crystallography and Biophysics, University of Madras, Guindy Campus, Chennai 600 025, India; b Department of Chemistry, Annamalai University, Annamalai Nagar 608 002, India; c Laboratory of X-ray Crystallography, Indian Institute of Chemical Technology, Hyderabad 500 007, India

## Abstract

In the title compound, C_22_H_23_N_3_O_2_S_2_, the five-membered ring is planar and the C_5_S ring adopts a chair conformation. The crystal packing is stabilized by inter­molecular N—H⋯O and C—H⋯O inter­actions, generating a chain and a centrosymmetric dimer, respectively.

## Related literature

For related literature, see: Allen *et al.* (1987 ▶); Isaac *et al.* (2003 ▶); Pan *et al.* (2003 ▶); Jung *et al.* (2004 ▶); Foroumadi *et al.* (2002 ▶); Jalilian *et al.* (2002 ▶); Leung-Toung *et al.* (2003 ▶); Schmidt *et al.* (1970 ▶); Cremer & Pople (1975 ▶); Nardelli (1983 ▶); Singh *et al.* (2003 ▶).
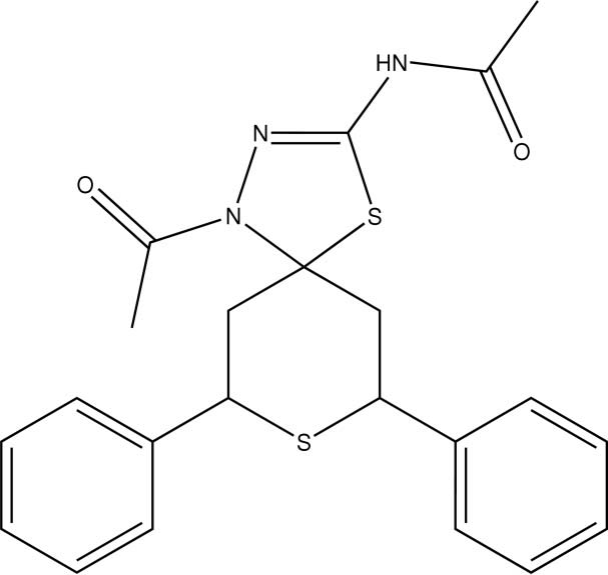


## Experimental

### 

#### Crystal data

C_22_H_23_N_3_O_2_S_2_
*M* *_r_* = 425.55
Monoclinic, 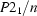

*a* = 12.3310 (7) Å
*b* = 16.0218 (9) Å
*c* = 12.3852 (7) Å
β = 116.714 (1)°
*V* = 2185.7 (2) Å^3^
*Z* = 4
Mo *K*α radiation
μ = 0.27 mm^−1^
*T* = 293 (2) K
0.25 × 0.24 × 0.22 mm

#### Data collection

Bruker SMART APEX CCD area-detector diffractometer
Absorption correction: none
24434 measured reflections
5139 independent reflections
4587 reflections with *I* > 2σ(*I*)
*R*_int_ = 0.020

#### Refinement

*R*[*F* ^2^ > 2σ(*F* ^2^)] = 0.038
*wR*(*F* ^2^) = 0.116
*S* = 0.97
5139 reflections
264 parameters
H-atom parameters constrained
Δρ_max_ = 0.31 e Å^−3^
Δρ_min_ = −0.17 e Å^−3^

### 

Data collection: *SMART* (Bruker, 2001 ▶); cell refinement: *SAINT* (Bruker, 2001 ▶); data reduction: *SAINT*; program(s) used to solve structure: *SHELXS97* (Sheldrick, 2008 ▶); program(s) used to refine structure: *SHELXL97* (Sheldrick, 2008 ▶); molecular graphics: *PLATON* (Spek, 2003 ▶); software used to prepare material for publication: *SHELXL97* and *PARST* (Nardelli, 1995 ▶).

## Supplementary Material

Crystal structure: contains datablocks I, global. DOI: 10.1107/S1600536808001025/at2527sup1.cif

Structure factors: contains datablocks I. DOI: 10.1107/S1600536808001025/at2527Isup2.hkl

Additional supplementary materials:  crystallographic information; 3D view; checkCIF report

## Figures and Tables

**Table 1:** Hydrogen-bond geometry (Å, °)

*D*—H⋯*A*	*D*—H	H⋯*A*	*D*⋯*A*	*D*—H⋯*A*
N3—H3⋯O1^i^	0.86	1.94	2.786 (2)	166
C5—H5⋯O2^ii^	0.98	2.49	3.446 (2)	163

Symmetry codes: (i) 
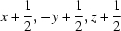
; (ii) 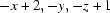.

**Fig. 1.**
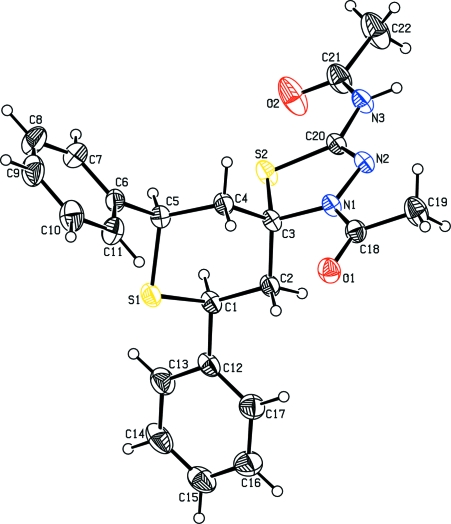
The molecular structure of (I), with atom labels and 30% probability displacement ellipsoids.

**Fig. 2.**
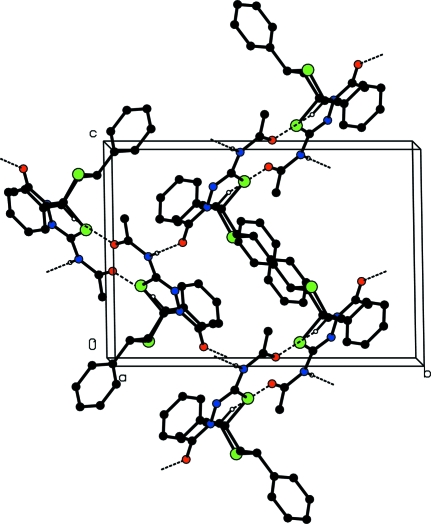
The packing of (I), viewed down the a axis, showing N—H···O and C—H···O intermolecular interactions. H atoms not involved in hydrogen bonding have been omitted.

## supplementary crystallographic information

### Comment

Tetrahydrothiopyrans play major roles in the field of medicinal chemistry (Isaac *et al.*, 2003). 1,3,4-Thiadiazoline nucleus, a biologically active heterocyclic ring, is also associated with a wide range of pharmacological activities (Pan *et al.*, 2003; Jung *et al.*, 2004; Foroumadi *et al.*, 2002; Jalilian *et al.*, 2002; Leung-Toung *et al.*, 2003). An essential component of the search for new leads in a drug-design programme is the synthesis of molecules, which is novel and resembles known biologically active molecules by virtue of the presence of certain pharmocophoric groups. Certain small heterocyclic molecules act as highly functionalized scaffolds and are pharmacophores of a number of biologically active and medicinally useful molecules. As the title compound (I) is of much biological importance, we have undertaken the crystal structure determination by X-ray diffraction.

The bond lengths and bond angles in (I) are comparable with those in the literature (Allen *et al.*, 1987). The sum of the bond angles around N1 atom [360.0 (3)°] indicates the *sp*^2^ hybridization. The torsion angles C19—C18—N1—N2 [-0.1 (2)°) and C19—C18—N1—C3 [-179.3 (1)°] indicate that atoms C18 and C19 lie in the plane of the five membered ring (N1/N2/C20/S2/C3). Also the torsion angles C22—C21—N3—C20 [-177.3 (2)°], O2—C21—N3—C20 [2.4 (3)°], C21—N3—C20—S2 [2.3 (2)°] and C21—N3—C20—N2 [-178.1 (2)°] indicate that the substituted moiety at C20 lie in the plane of the ring to which it is attached. The dihedral angle between the two phenyl rings in the structure is about 77.6 (1)° which clearly indicates that the two phenyl rings are nearly perpendicular to each other.

The six membered ring C1—C5/S1 adopts chair conformation with the puckering parameters (Cremer & Pople, 1975) and the smallest displacement asymmetry parameters (Nardelli, 1983) being q_2_ = 0.117 (1) Å, q_3_ = 0.651 (1) Å; Q_T_ = 0.661 (1)Å and θ = 10.2 (1)°.

The crystal packing is stabilized N—H···O and C—H···O intermolecular interaction generating a chain of C(7) and a centrosymmetric dimer of *R*_2_^2^(18) ring, respectively.

### Experimental

2,6-Diphenyltetrahydrothiopyran-4-one thiosemicarbazone (0.025 mol) was treated with freshly distilled acetic anhydride and the mixture was refluxed for 8 h on a water bath (363–373 K). The removal of solvent from the cooled reaction mixture in vaccuo afforded 4-acetyl-2-acetylamino-5-spiro-((*r*)-2,(*c*)6-diphenyltetrahydrothiopyran-4-yl)- 4,5-dihydro-[1,3,4]thiadiazole which was purified in neutral alumina column using n-hexane-ethyl acetate (4:1) as eluent. The pure compound was recrystallized from ethanol [m.p.: 399 K].

### Refinement

All H-atoms were refined using a riding model with d(C—H) = 0.93 Å, *U*_iso_=1.2*U*_eq_ (C) for aromatic, 0.98 Å, *U*_iso_ = 1.2*U*_eq_ (C) for CH, 0.97 Å, *U*_iso_ = 1.2*U*_eq_ (C) for CH_2_, 0.96 Å, *U*_iso_ = 1.5*U*_eq_ (C) for CH_3_ atoms and 0.86 Å, *U*_iso_ = 1.2*U*_eq_ (N) for the NH group.

### Crystal data

**Table:**

C_22_H_23_N_3_O_2_S_2_	*F*_000_ = 896
*M**_r_* = 425.55	*D*_x_ = 1.293 Mg m^−^^3^
Monoclinic, *P*2_1_/*n*	Mo *K*α radiation λ = 0.71073 Å
Hall symbol: -P 2yn	Cell parameters from 2504 reflections
*a* = 12.3310 (7) Å	θ = 1.9–28.0º
*b* = 16.0218 (9) Å	µ = 0.27 mm^−^^1^
*c* = 12.3852 (7) Å	*T* = 293 (2) K
β = 116.714 (1)º	Block, colourless
*V* = 2185.7 (2) Å^3^	0.25 × 0.24 × 0.22 mm
*Z* = 4	

### Data collection

**Table:**

Bruker SMART APEX CCD area-detector diffractometer	4587 reflections with *I* > 2σ(*I*)
Radiation source: fine-focus sealed tube	*R*_int_ = 0.020
Monochromator: graphite	θ_max_ = 28.0º
*T* = 293(2) K	θ_min_ = 1.9º
ω scans	*h* = −15→15
Absorption correction: none	*k* = −21→21
24434 measured reflections	*l* = −16→16
5139 independent reflections	

### Refinement

**Table:**

Refinement on *F*^2^	Secondary atom site location: difference Fourier map
Least-squares matrix: full	Hydrogen site location: inferred from neighbouring sites
*R*[*F*^2^ > 2σ(*F*^2^)] = 0.038	H-atom parameters constrained
*wR*(*F*^2^) = 0.116	*w* = 1/[σ^2^(*F*_o_^2^) + (0.0759*P*)^2^ + 0.4937*P*] where *P* = (*F*_o_^2^ + 2*F*_c_^2^)/3
*S* = 0.97	(Δ/σ)_max_ = 0.031
5139 reflections	Δρ_max_ = 0.31 e Å^−^^3^
264 parameters	Δρ_min_ = −0.17 e Å^−^^3^
Primary atom site location: structure-invariant direct methods	Extinction correction: none

### Special details

**Table:**

Geometry. All e.s.d.'s (except the e.s.d. in the dihedral angle between two l.s. planes) are estimated using the full covariance matrix. The cell e.s.d.'s are taken into account individually in the estimation of e.s.d.'s in distances, angles and torsion angles; correlations between e.s.d.'s in cell parameters are only used when they are defined by crystal symmetry. An approximate (isotropic) treatment of cell e.s.d.'s is used for estimating e.s.d.'s involving l.s. planes.
Refinement. Refinement of *F*^2^ against ALL reflections. The weighted *R*-factor *wR* and goodness of fit *S* are based on *F*^2^, conventional *R*-factors *R* are based on *F*, with *F* set to zero for negative *F*^2^. The threshold expression of *F*^2^ > σ(*F*^2^) is used only for calculating *R*-factors(gt) *etc*. and is not relevant to the choice of reflections for refinement. *R*-factors based on *F*^2^ are statistically about twice as large as those based on *F*, and *R*- factors based on ALL data will be even larger.

### Fractional atomic coordinates and isotropic or equivalent isotropic displacement parameters (Å^2^)

**Table:**

	*x*	*y*	*z*	*U*_iso_*/*U*_eq_	
C1	0.81098 (11)	0.05407 (8)	0.11215 (12)	0.0408 (3)	
H1	0.8386	0.0188	0.1844	0.049*	
C2	0.91557 (11)	0.11046 (8)	0.12512 (12)	0.0409 (3)	
H2A	0.8872	0.1490	0.0575	0.049*	
H2B	0.9794	0.0766	0.1221	0.049*	
C3	0.96801 (10)	0.16028 (8)	0.24349 (11)	0.0377 (3)	
C4	0.87477 (11)	0.21761 (8)	0.25620 (12)	0.0405 (3)	
H4A	0.9135	0.2456	0.3338	0.049*	
H4B	0.8499	0.2601	0.1938	0.049*	
C5	0.76159 (11)	0.17236 (8)	0.24675 (12)	0.0403 (3)	
H5	0.7863	0.1315	0.3124	0.048*	
C6	0.67241 (12)	0.23270 (8)	0.25743 (12)	0.0418 (3)	
C7	0.64638 (16)	0.22878 (12)	0.35475 (15)	0.0586 (4)	
H7	0.6834	0.1886	0.4143	0.070*	
C8	0.5648 (2)	0.28496 (14)	0.36371 (19)	0.0752 (5)	
H8	0.5471	0.2818	0.4291	0.090*	
C9	0.51035 (17)	0.34484 (12)	0.2773 (2)	0.0690 (5)	
H9	0.4571	0.3827	0.2850	0.083*	
C10	0.53404 (16)	0.34912 (11)	0.17975 (19)	0.0632 (4)	
H10	0.4963	0.3893	0.1203	0.076*	
C11	0.61498 (15)	0.29289 (10)	0.17013 (16)	0.0538 (4)	
H11	0.6309	0.2958	0.1036	0.065*	
C12	0.76348 (11)	−0.00201 (8)	0.00198 (13)	0.0448 (3)	
C13	0.68438 (16)	−0.06649 (11)	−0.00790 (18)	0.0625 (4)	
H13	0.6616	−0.0749	0.0534	0.075*	
C14	0.63888 (17)	−0.11863 (11)	−0.1084 (2)	0.0719 (5)	
H14	0.5863	−0.1617	−0.1135	0.086*	
C15	0.67076 (16)	−0.10715 (11)	−0.19984 (19)	0.0681 (5)	
H15	0.6392	−0.1416	−0.2675	0.082*	
C16	0.74988 (18)	−0.04419 (12)	−0.19032 (18)	0.0693 (5)	
H16	0.7728	−0.0364	−0.2516	0.083*	
C17	0.79604 (16)	0.00785 (10)	−0.09063 (15)	0.0578 (4)	
H17	0.8498	0.0501	−0.0857	0.069*	
C18	1.07172 (12)	0.27042 (8)	0.17789 (12)	0.0411 (3)	
C19	1.18800 (14)	0.31368 (11)	0.20301 (17)	0.0606 (4)	
H19A	1.2396	0.2764	0.1865	0.091*	
H19B	1.2279	0.3303	0.2863	0.091*	
H19C	1.1711	0.3621	0.1524	0.091*	
C20	1.17574 (11)	0.13205 (8)	0.41606 (11)	0.0382 (3)	
C21	1.28104 (14)	0.04085 (11)	0.58940 (16)	0.0602 (4)	
C22	1.40213 (17)	0.02732 (16)	0.6959 (2)	0.0910 (8)	
H22A	1.3910	0.0001	0.7593	0.137*	
H22B	1.4412	0.0802	0.7244	0.137*	
H22C	1.4517	−0.0070	0.6723	0.137*	
N1	1.07548 (9)	0.21007 (7)	0.25605 (9)	0.0385 (2)	
N2	1.18656 (9)	0.19210 (7)	0.35415 (10)	0.0391 (2)	
N3	1.27755 (10)	0.10526 (8)	0.51668 (10)	0.0459 (3)	
H3	1.3443	0.1316	0.5348	0.055*	
S1	0.68373 (3)	0.11763 (2)	0.10259 (3)	0.04552 (11)	
S2	1.03376 (3)	0.08660 (2)	0.37248 (3)	0.04760 (12)	
O1	0.97614 (9)	0.28782 (7)	0.08854 (9)	0.0496 (2)	
O2	1.19260 (12)	−0.00139 (10)	0.56887 (15)	0.0911 (5)	

### Atomic displacement parameters (Å^2^)

**Table:**

	*U*^11^	*U*^22^	*U*^33^	*U*^12^	*U*^13^	*U*^23^
C1	0.0315 (6)	0.0393 (6)	0.0463 (6)	−0.0006 (5)	0.0128 (5)	−0.0023 (5)
C2	0.0295 (6)	0.0440 (7)	0.0462 (7)	−0.0014 (5)	0.0144 (5)	−0.0066 (5)
C3	0.0275 (5)	0.0391 (6)	0.0415 (6)	−0.0027 (4)	0.0111 (5)	−0.0001 (5)
C4	0.0334 (6)	0.0417 (6)	0.0454 (6)	−0.0017 (5)	0.0169 (5)	−0.0033 (5)
C5	0.0356 (6)	0.0420 (6)	0.0441 (6)	−0.0007 (5)	0.0187 (5)	0.0010 (5)
C6	0.0362 (6)	0.0436 (6)	0.0495 (7)	−0.0049 (5)	0.0228 (5)	−0.0037 (5)
C7	0.0614 (9)	0.0688 (10)	0.0551 (8)	−0.0016 (8)	0.0347 (8)	−0.0018 (7)
C8	0.0779 (12)	0.0931 (14)	0.0782 (12)	−0.0035 (11)	0.0560 (11)	−0.0175 (11)
C9	0.0560 (9)	0.0644 (10)	0.1010 (14)	−0.0014 (8)	0.0481 (10)	−0.0211 (10)
C10	0.0539 (9)	0.0514 (9)	0.0889 (12)	0.0088 (7)	0.0362 (9)	0.0020 (8)
C11	0.0528 (8)	0.0531 (8)	0.0657 (9)	0.0082 (6)	0.0358 (7)	0.0066 (7)
C12	0.0320 (6)	0.0397 (6)	0.0537 (7)	0.0014 (5)	0.0113 (5)	−0.0061 (5)
C13	0.0533 (9)	0.0579 (9)	0.0740 (10)	−0.0153 (7)	0.0267 (8)	−0.0141 (8)
C14	0.0498 (9)	0.0556 (10)	0.0958 (14)	−0.0159 (7)	0.0200 (9)	−0.0223 (9)
C15	0.0498 (9)	0.0622 (10)	0.0776 (11)	0.0029 (7)	0.0156 (8)	−0.0305 (9)
C16	0.0708 (11)	0.0702 (11)	0.0666 (10)	−0.0030 (9)	0.0305 (9)	−0.0223 (9)
C17	0.0576 (9)	0.0522 (8)	0.0635 (9)	−0.0072 (7)	0.0272 (8)	−0.0143 (7)
C18	0.0370 (6)	0.0392 (6)	0.0463 (6)	0.0018 (5)	0.0180 (5)	0.0003 (5)
C19	0.0461 (8)	0.0560 (9)	0.0721 (10)	−0.0088 (7)	0.0198 (7)	0.0140 (7)
C20	0.0289 (5)	0.0389 (6)	0.0415 (6)	−0.0018 (4)	0.0111 (5)	−0.0034 (5)
C21	0.0413 (7)	0.0576 (9)	0.0678 (10)	0.0010 (6)	0.0121 (7)	0.0199 (7)
C22	0.0484 (9)	0.1010 (16)	0.0918 (14)	−0.0008 (10)	0.0032 (9)	0.0505 (13)
N1	0.0269 (5)	0.0415 (5)	0.0417 (5)	−0.0022 (4)	0.0107 (4)	0.0011 (4)
N2	0.0278 (5)	0.0422 (5)	0.0402 (5)	−0.0023 (4)	0.0089 (4)	−0.0015 (4)
N3	0.0308 (5)	0.0494 (6)	0.0463 (6)	−0.0035 (4)	0.0073 (4)	0.0060 (5)
S1	0.02991 (17)	0.0501 (2)	0.0527 (2)	−0.00154 (12)	0.01512 (14)	−0.00724 (14)
S2	0.03201 (17)	0.0476 (2)	0.0523 (2)	−0.00652 (12)	0.00926 (14)	0.00873 (14)
O1	0.0386 (5)	0.0543 (6)	0.0509 (5)	0.0070 (4)	0.0156 (4)	0.0104 (4)
O2	0.0511 (7)	0.0829 (9)	0.1081 (11)	−0.0123 (6)	0.0081 (7)	0.0488 (8)

### Geometric parameters (Å, °)

**Table:**

C1—C12	1.5145 (18)	C12—C13	1.388 (2)
C1—C2	1.5228 (17)	C13—C14	1.391 (3)
C1—S1	1.8292 (13)	C13—H13	0.9300
C1—H1	0.9800	C14—C15	1.368 (3)
C2—C3	1.5337 (17)	C14—H14	0.9300
C2—H2A	0.9700	C15—C16	1.371 (3)
C2—H2B	0.9700	C15—H15	0.9300
C3—N1	1.4937 (15)	C16—C17	1.383 (2)
C3—C4	1.5341 (17)	C16—H16	0.9300
C3—S2	1.8543 (13)	C17—H17	0.9300
C4—C5	1.5297 (17)	C18—O1	1.2324 (16)
C4—H4A	0.9700	C18—N1	1.3541 (17)
C4—H4B	0.9700	C18—C19	1.4941 (19)
C5—C6	1.5140 (18)	C19—H19A	0.9600
C5—S1	1.8268 (13)	C19—H19B	0.9600
C5—H5	0.9800	C19—H19C	0.9600
C6—C11	1.383 (2)	C20—N2	1.2742 (17)
C6—C7	1.3811 (19)	C20—N3	1.3808 (16)
C7—C8	1.391 (3)	C20—S2	1.7427 (12)
C7—H7	0.9300	C21—O2	1.209 (2)
C8—C9	1.368 (3)	C21—N3	1.3577 (19)
C8—H8	0.9300	C21—C22	1.499 (2)
C9—C10	1.366 (3)	C22—H22A	0.9600
C9—H9	0.9300	C22—H22B	0.9600
C10—C11	1.389 (2)	C22—H22C	0.9600
C10—H10	0.9300	N1—N2	1.3924 (14)
C11—H11	0.9300	N3—H3	0.8600
C12—C17	1.384 (2)		
			
C12—C1—C2	114.40 (11)	C17—C12—C13	117.78 (14)
C12—C1—S1	107.39 (8)	C17—C12—C1	122.70 (12)
C2—C1—S1	109.74 (9)	C13—C12—C1	119.52 (14)
C12—C1—H1	108.4	C12—C13—C14	120.71 (17)
C2—C1—H1	108.4	C12—C13—H13	119.6
S1—C1—H1	108.4	C14—C13—H13	119.6
C1—C2—C3	112.51 (11)	C15—C14—C13	120.66 (16)
C1—C2—H2A	109.1	C15—C14—H14	119.7
C3—C2—H2A	109.1	C13—C14—H14	119.7
C1—C2—H2B	109.1	C14—C15—C16	119.07 (16)
C3—C2—H2B	109.1	C14—C15—H15	120.5
H2A—C2—H2B	107.8	C16—C15—H15	120.5
N1—C3—C4	109.89 (10)	C15—C16—C17	120.78 (18)
N1—C3—C2	110.53 (10)	C15—C16—H16	119.6
C4—C3—C2	113.35 (10)	C17—C16—H16	119.6
N1—C3—S2	103.09 (8)	C16—C17—C12	120.99 (16)
C4—C3—S2	110.51 (9)	C16—C17—H17	119.5
C2—C3—S2	108.99 (9)	C12—C17—H17	119.5
C5—C4—C3	114.11 (10)	O1—C18—N1	121.01 (12)
C5—C4—H4A	108.7	O1—C18—C19	121.52 (13)
C3—C4—H4A	108.7	N1—C18—C19	117.46 (12)
C5—C4—H4B	108.7	C18—C19—H19A	109.5
C3—C4—H4B	108.7	C18—C19—H19B	109.5
H4A—C4—H4B	107.6	H19A—C19—H19B	109.5
C6—C5—C4	111.36 (11)	C18—C19—H19C	109.5
C6—C5—S1	107.98 (9)	H19A—C19—H19C	109.5
C4—C5—S1	111.28 (9)	H19B—C19—H19C	109.5
C6—C5—H5	108.7	N2—C20—N3	118.63 (11)
C4—C5—H5	108.7	N2—C20—S2	119.44 (9)
S1—C5—H5	108.7	N3—C20—S2	121.93 (10)
C11—C6—C7	118.41 (14)	O2—C21—N3	121.95 (14)
C11—C6—C5	120.91 (12)	O2—C21—C22	123.48 (16)
C7—C6—C5	120.67 (13)	N3—C21—C22	114.57 (14)
C6—C7—C8	120.03 (17)	C21—C22—H22A	109.5
C6—C7—H7	120.0	C21—C22—H22B	109.5
C8—C7—H7	120.0	H22A—C22—H22B	109.5
C9—C8—C7	120.65 (16)	C21—C22—H22C	109.5
C9—C8—H8	119.7	H22A—C22—H22C	109.5
C7—C8—H8	119.7	H22B—C22—H22C	109.5
C8—C9—C10	120.14 (16)	C18—N1—N2	118.39 (10)
C8—C9—H9	119.9	C18—N1—C3	124.27 (10)
C10—C9—H9	119.9	N2—N1—C3	117.33 (10)
C9—C10—C11	119.39 (17)	C20—N2—N1	110.68 (10)
C9—C10—H10	120.3	C21—N3—C20	125.43 (12)
C11—C10—H10	120.3	C21—N3—H3	117.3
C6—C11—C10	121.36 (15)	C20—N3—H3	117.3
C6—C11—H11	119.3	C5—S1—C1	98.36 (6)
C10—C11—H11	119.3	C20—S2—C3	89.43 (6)
			
C12—C1—C2—C3	−174.88 (10)	C15—C16—C17—C12	−0.2 (3)
S1—C1—C2—C3	64.36 (12)	C13—C12—C17—C16	0.9 (2)
C1—C2—C3—N1	176.11 (10)	C1—C12—C17—C16	−179.42 (15)
C1—C2—C3—C4	−60.01 (14)	O1—C18—N1—N2	178.81 (12)
C1—C2—C3—S2	63.50 (12)	C19—C18—N1—N2	−0.07 (18)
N1—C3—C4—C5	−179.06 (10)	O1—C18—N1—C3	−0.5 (2)
C2—C3—C4—C5	56.72 (14)	C19—C18—N1—C3	−179.34 (13)
S2—C3—C4—C5	−65.95 (12)	C4—C3—N1—C18	−64.69 (15)
C3—C4—C5—C6	−178.82 (11)	C2—C3—N1—C18	61.15 (15)
C3—C4—C5—S1	−58.29 (13)	S2—C3—N1—C18	177.50 (10)
C4—C5—C6—C11	65.69 (17)	C4—C3—N1—N2	116.04 (12)
S1—C5—C6—C11	−56.76 (15)	C2—C3—N1—N2	−118.12 (11)
C4—C5—C6—C7	−114.40 (15)	S2—C3—N1—N2	−1.78 (12)
S1—C5—C6—C7	123.16 (13)	N3—C20—N2—N1	179.47 (11)
C11—C6—C7—C8	−0.5 (2)	S2—C20—N2—N1	−0.92 (15)
C5—C6—C7—C8	179.61 (16)	C18—N1—N2—C20	−177.50 (11)
C6—C7—C8—C9	−0.5 (3)	C3—N1—N2—C20	1.82 (15)
C7—C8—C9—C10	1.2 (3)	O2—C21—N3—C20	2.4 (3)
C8—C9—C10—C11	−0.8 (3)	C22—C21—N3—C20	−177.33 (18)
C7—C6—C11—C10	0.8 (2)	N2—C20—N3—C21	−178.11 (15)
C5—C6—C11—C10	−179.28 (14)	S2—C20—N3—C21	2.3 (2)
C9—C10—C11—C6	−0.2 (3)	C6—C5—S1—C1	178.14 (9)
C2—C1—C12—C17	−11.55 (19)	C4—C5—S1—C1	55.64 (10)
S1—C1—C12—C17	110.50 (14)	C12—C1—S1—C5	176.47 (9)
C2—C1—C12—C13	168.17 (13)	C2—C1—S1—C5	−58.62 (10)
S1—C1—C12—C13	−69.78 (15)	N2—C20—S2—C3	−0.11 (11)
C17—C12—C13—C14	−0.6 (2)	N3—C20—S2—C3	179.49 (11)
C1—C12—C13—C14	179.65 (15)	N1—C3—S2—C20	0.98 (8)
C12—C13—C14—C15	−0.3 (3)	C4—C3—S2—C20	−116.40 (9)
C13—C14—C15—C16	1.0 (3)	C2—C3—S2—C20	118.42 (9)
C14—C15—C16—C17	−0.8 (3)		

### Hydrogen-bond geometry (Å, °)

**Table:**

*D*—H···*A*	*D*—H	H···*A*	*D*···*A*	*D*—H···*A*
N3—H3···O1^i^	0.86	1.94	2.786 (2)	166
C5—H5···O2^ii^	0.98	2.49	3.446 (2)	163

Symmetry codes: (i) *x*+1/2, −*y*+1/2, *z*+1/2; (ii) −*x*+2, −*y*, −*z*+1.
